# Supplementary material for: Using surgical wrapping material for the fabrication of respirator masks
Source: PLoS One. 2020 Jul 21;15(7):e0236239. doi: 10.1371/journal.pone.0236239 (PMC7373262; doi:10.1371/journal.pone.0236239)
Supplement: S1 Data — (PDF) [file pone.0236239.s001.pdf]

Filtration efficiency test

				0,3µm	0,5µm	3,0µm
**Unsterilised material (from package)**	White layer only	Sample 1 (n = 4)	3/4/2020	46.40%	68.44%	92.38%
		Sample 2 (n = 4)	3/4/2020	51.13%	73.49%	93.41%
			**Averaged**	**48.76%**	**70.97%**	**92.89%**
			**Intersample Standard Dev.**	**3.34%**	**3.58%**	**0.73%**
	Blue layer only	Sample 3 (n = 4)	3/4/2020	48.37%	68.97%	92.87%
		Sample 4 (n = 4)	3/4/2020	49.91%	70.65%	93.90%
			**Averaged**	**49.14%**	**69.81%**	**93.39%**
			**Intersample Standard Dev.**	**1.09%**	**1.19%**	**0.73%**
	Single Layer	Sample 5(n = 4)	3/4/2020	70.42%	90.17%	99.65%
		Sample 6 (n = 4)	3/4/2020	69.74%	89.19%	99.84%
			**Averaged**	**70.08%**	**89.68%**	**99.74%**
			**Intersample Standard Dev.**	**0.48%**	**0.70%**	**0.13%**
	Double Layer	Sample 7 (n = 4)	3/4/2020	87.99%	98.35%	99.98%
		Sample 8 (n = 4)	3/4/2020	87.38%	98.21%	99.98%
		Sample 53 (n = 4)	3/4/2020	84.42%	97.50%	99.96%
			**Averaged**	**86.60%**	**98.02%**	**99.97%**
			**Intersample Standard Dev.**	**1.91%**	**0.46%**	**0.01%**
	Double Layer, reversed	Sample 9 (n = 4)	3/4/2020	85.35%	97.79%	99.96%
		Sample 10 (n = 4)	3/4/2020	83.95%	97.34%	100.00%
			**Averaged**	**84.65%**	**97.57%**	**99.98%**
			**Intersample Standard Dev.**	**0.99%**	**0.32%**	**0.03%**

	Double Layer, after use 20 minutes	Sample 11 (n = 4)	3/4/2020	87.64%	98.64%	100.00%
		Sample 12 (n = 4)	3/4/2020	87.54%	98.63%	100.00%
			**Averaged**	**87.59%**	**98.63%**	**100.00%**
			**Intersample Standard Dev.**	**0.07%**	**0.00%**	**0.00%**
	Triple layer	Sample 13 (n = 4)	3/4/2020	94.10%	99.51%	99.98%
		Sample 14 (n = 4)	3/4/2020	93.58%	99.39%	100.00%
			**Averaged**	**93.84%**	**99.45%**	**99.99%**
			**Intersample Standard Dev.**	**0.37%**	**0.08%**	**0.01%**
**Steam Sterilised (15min, 121 degrees Celsius, 2 atm)**	Single Layer, 1x sterilized	Sample 15 (n = 4)	3/4/2020	66.15%	87.14%	99.40%
		Sample 16 (n = 4)	3/4/2020	66.58%	87.24%	99.73%
			**Averaged**	**66.37%**	**87.19%**	**99.57%**
			**Intersample Standard Dev.**	**0.31%**	**0.07%**	**0.23%**
	Double Layer, 1x sterilized	Sample 17 (n = 4)	3/4/2020	80.63%	95.91%	100.00%
		Sample 18 (n = 4)	3/4/2020	80.16%	95.67%	99.98%
			**Averaged**	**80.39%**	**95.79%**	**99.99%**
			**Intersample Standard Dev.**	**0.33%**	**0.17%**	**0.01%**
	Triple layer, 1x sterilized	Sample 19 (n = 4)	3/4/2020	87.98%	98.35%	99.96%
		Sample 20 (n = 4)	3/4/2020	88.57%	98.52%	99.98%
		Sample 21 (n = 4)	12/5/2020	82.74%	98.94%	99.91%
		Sample 22 (n = 4)	12/5/2020	83.71%	99.02%	99.97%
		Sample 23 (n = 4)	12/5/2020	83.77%	98.98%	99.99%
		Sample 24 (n = 4)	12/5/2020	83.91%	98.95%	99.97%
			**Averaged**	**85.11%**	**98.44%**	**99.97%**
			**Intersample Standard Dev.**	**2.49%**	**0.28%**	**0.03%**

Triple layer, 2x sterilized	Sample 25 (n = 4)	12/5/2020	89.08%	99.52%	99.97%
	Sample 26 (n = 4)	12/5/2020	86.52%	99.26%	99.93%
	Sample 27 (n = 4)	12/5/2020	87.97%	99.42%	99.97%
	Sample 28 (n = 4)	12/5/2020	86.51%	99.31%	99.96%
		**Averaged**	**87.52%**	**99.38%**	**99.96%**
		**Intersample Standard Dev.**	**1.25%**	**0.12%**	**0.02%**
Triple layer, 3x sterilized	Sample 29 (n = 4)	12/5/2020	81.71%	98.74%	99.97%
	Sample 30 (n = 4)	12/5/2020	82.88%	98.94%	99.92%
	Sample 31 (n = 4)	12/5/2020	83.66%	99.01%	99.97%
	Sample 32 (n = 4)	12/5/2020	82.07%	98.80%	99.99%
		**Averaged**	**82.58%**	**98.87%**	**99.97%**
		**Intersample Standard Dev.**	**0.87%**	**0.12%**	**0.03%**
Triple layer, 4x sterilized	Sample 33 (n = 4)	12/5/2020	86.00%	99.27%	99.96%
	Sample 34 (n = 4)	12/5/2020	86.36%	99.29%	99.90%
	Sample 35 (n = 4)	12/5/2020	85.60%	99.21%	99.97%
	Sample 36 (n = 4)	12/5/2020	85.27%	99.17%	99.92%
		**Averaged**	**85.81%**	**99.23%**	**99.94%**
		**Intersample Standard Dev.**	**0.47%**	**0.05%**	**0.03%**
Triple layer, 5x sterilized	Sample 37 (n = 4)	12/5/2020	83.61%	99.00%	99.95%
	Sample 38 (n = 4)	12/5/2020	84.71%	99.16%	99.98%
	Sample 39 (n = 4)	12/5/2020	83.19%	98.98%	99.96%
	Sample 40 (n = 4)	12/5/2020	82.42%	98.88%	99.99%
		**Averaged**	**83.48%**	**99.00%**	**99.97%**
		**Intersample Standard Dev.**	**0.96%**	**0.11%**	**0.02%**

**Comparison with commercial Masks & Respirators**	Surgical Mask	Sample 41	12/5/2020	55.89%	89.26%	98.96%
		Sample 42	12/5/2020	51.48%	87.47%	98.01%
		Sample 43	12/5/2020	57.64%	89.86%	99.48%
		Sample 44	12/5/2020	53.07%	87.84%	99.22%
			**Averaged**	**54.52%**	**88.61%**	**98.92%**
			**Intersample Standard Dev.**	**2.77%**	**1.13%**	**0.64%**
	Disposable Face Mask FFP2	Sample 45	12/5/2020	93.55%	99.52%	100.00%
		Sample 46	12/5/2020	93.93%	99.58%	100.00%
		Sample 47	12/5/2020	94.35%	99.61%	100.00%
		Sample 48	12/5/2020	94.47%	99.57%	100.00%
			**Averaged**	**94.08%**	**99.57%**	**100.00%**
			**Intersample Standard Dev.**	**0.42%**	**0.04%**	**0.00%**
	3M 8320 FFP2 NR D Mask	Sample 49	12/5/2020	98.91%	99.90%	99.91%
		Sample 50	12/5/2020	95.91%	99.65%	100.00%
		Sample 51	12/5/2020	98.10%	99.78%	100.00%
		Sample 52	12/5/2020	96.84%	99.65%	99.83%
			**Averaged**	**97.44%**	**99.75%**	**99.94%**
			**Intersample Standard Dev.**	**1.33%**	**0.12%**	**0.08%**
