# Supplementary material for: Using surgical wrapping material for the fabrication of respirator masks
Source: PLoS One. 2020 Jul 21;15(7):e0236239. doi: 10.1371/journal.pone.0236239 (PMC7373262; doi:10.1371/journal.pone.0236239)
Supplement: S2 Data — (PDF) [file pone.0236239.s002.pdf]

# Halyard Quickcheck H300 material

| Sample # | Code | Description | 0,3µm | 0,5µm | 3µm |
|---|---|---|---|---|---|
| **Background Measurements #1** | | | 382237 | 148794 | 1131 |
| | | | 384541 | 150992 | 1351 |
| | | | 381320 | 149019 | 1219 |
| | | | 379900 | 149255 | 1172 |
| | | **Average particles** | **382000** | **149515** | **1218** |
| **Sample 1** | 03042020-CSDr | Sample 1, White layer only of Halyard Quickcheck H300 | 149736 | 31990 | 43 |
| | 03042020-CSDr | *Background Measurements #2 used for calculation of filter efficiency* | 149779 | 32265 | 59 |
| | 03042020-CSDr | | 150152 | 32037 | 64 |
| | 03042020-CSDr | | 148730 | 31759 | 34 |
| | | **Average particles** | **149599** | **32013** | **50** |
| | | **% Filter collection efficiency** | **46.40%** | **68.44%** | **92.38%** |
| **Sample 2** | 03042020-CSDs | Sample 2, White layer only of Halyard Quickcheck H300 | 137656 | 27094 | 41 |
| | 03042020-CSDs | *Background Measurements #2 used for calculation of filter efficiency* | 136845 | 26952 | 45 |
| | 03042020-CSDs | | 135309 | 26686 | 55 |
| | 03042020-CSDs | | 135784 | 26802 | 32 |
| | | **Average particles** | **136399** | **26884** | **43** |
| | | **% Filter collection efficiency** | **51.13%** | **73.49%** | **93.41%** |
| **Sample 3** | 03042020-CSDt | Sample 3, Blue layer only of Halyard Quickcheck H300 | 145796 | 31630 | 57 |
| | 03042020-CSDt | *Background Measurements #2 used for calculation of filter efficiency* | 144838 | 31698 | 44 |
| | 03042020-CSDt | | 143250 | 31574 | 47 |
| | 03042020-CSDt | | 142487 | 30974 | 39 |
| | | **Average particles** | **144093** | **31469** | **47** |
| | | **% Filter collection efficiency** | **48.37%** | **68.97%** | **92.87%** |

| | | | | | |
|---|---|---|---|---|---|
| **Sample 4** | 03042020-CSDu | Sample 4, Blue layer only of Halyard Quickcheck H300 | 140824 | 29824 | 44 |
| | 03042020-CSDu | *Background Measurements #2 used for calculation of filter efficiency* | 140301 | 29847 | 35 |
| | 03042020-CSDu | | 137981 | 29649 | 52 |
| | 03042020-CSDu | | 140129 | 29740 | 29 |
| | | **Average particles** | **139809** | **29765** | **40** |
| | | **% Filter collection efficiency** | **49.91%** | **70.65%** | **93.90%** |
| **Sample 5** | 03042020-CSDc | Sample 5, Single layer Halyard QuickCheck H300 | 112157 | 14339 | 7 |
| | 03042020-CSDc | *Background Measurements #1 used for calculation of filter efficiency* | 112933 | 14682 | 5 |
| | 03042020-CSDc | | 113143 | 14911 | 1 |
| | 03042020-CSDc | | 113738 | 14837 | 4 |
| | | **Average particles** | **112993** | **14692** | **4** |
| | | **% Filter collection efficiency** | **70.42%** | **90.17%** | **99.65%** |
| **Sample 6** | 03042020-CSDd | Sample 6, Single layer Halyard QuickCheck H300 | 116547 | 16297 | 1 |
| | 03042020-CSDd | *Background Measurements #1 used for calculation of filter efficiency* | 115368 | 16135 | 0 |
| | 03042020-CSDd | | 115165 | 15847 | 5 |
| | 03042020-CSDd | | 115297 | 16372 | 2 |
| | | **Average particles** | **115594** | **16163** | **2** |
| | | **% Filter collection efficiency** | **69.74%** | **89.19%** | **99.84%** |
| **Sample 7** | 03042020-CSDa | Sample 7, Double layer Halyard QuickCheck H300 | 45893 | 2329 | 1 |
| | 03042020-CSDa | *Background Measurements #1 used for calculation of filter efficiency* | 46009 | 2458 | 0 |
| | 03042020-CSDa | | 45822 | 2512 | 0 |
| | 03042020-CSDa | | 45771 | 2595 | 0 |
| | | **Average particles** | **45874** | **2474** | **0** |
| | | **% Filter collection efficiency** | **87.99%** | **98.35%** | **99.98%** |
| **Sample 8** | 03042020-CSDb | Sample 8, Double layer Halyard QuickCheck H300 | 48265 | 2642 | 1 |
| | 03042020-CSDb | *Background Measurements #1 used for calculation of filter efficiency* | 47795 | 2688 | 0 |
| | 03042020-CSDb | | 48247 | 2683 | 0 |
| | 03042020-CSDb | | 48553 | 2691 | 0 |
| | | **Average particles** | **48215** | **2676** | **0** |
| | | **% Filter collection efficiency** | **87.38%** | **98.21%** | **99.98%** |

| | | | | | |
|---|---|---|---|---|---|
| **Sample 9** | 03042020-CSDn | Sample 9, Double layer Halyard QuickCheck H300 - reversed | 41124 | 2228 | 0 |
| | 03042020-CSDn | *Background Measurements #2 used for calculation of filter efficiency* | 40872 | 2192 | 0 |
| | 03042020-CSDn | | 40643 | 2246 | 0 |
| | 03042020-CSDn | | 40963 | 2285 | 1 |
| | | **Average particles** | **40901** | **2238** | **0** |
| | | **% Filter collection efficiency** | **85.35%** | **97.79%** | **99.96%** |
| **Sample 10** | 03042020-CSDo | Sample 10, Double layer Halyard QuickCheck H300 - reversed | 44807 | 2671 | 0 |
| | 03042020-CSDo | *Background Measurements #2 used for calculation of filter efficiency* | 45197 | 2677 | 0 |
| | 03042020-CSDo | | 45079 | 2797 | 0 |
| | 03042020-CSDo | | 44115 | 2640 | 0 |
| | | **Average particles** | **44800** | **2696** | **0** |
| | | **% Filter collection efficiency** | **83.95%** | **97.34%** | **100.00%** |
| **Sample 11** | 03042020-CSDv | Sample 11, Double Layer Halyard Quickcheck H300 after 20 min wearing | 34432 | 1365 | 0 |
| | 03042020-CSDv | *Background Measurements #2 used for calculation of filter efficiency* | 34240 | 1422 | 0 |
| | 03042020-CSDv | | 34764 | 1369 | 0 |
| | 03042020-CSDv | | 34545 | 1375 | 0 |
| | | **Average particles** | **34495** | **1383** | **0** |
| | | **% Filter collection efficiency** | **87.64%** | **98.64%** | **100.00%** |
| **Sample 12** | 03042020-CSDv | Sample 12, Double Layer Halyard Quickcheck H300 after 20 min wearing | 34870 | 1401 | 0 |
| | 03042020-CSDv | *Background Measurements #2 used for calculation of filter efficiency* | 35096 | 1390 | 0 |
| | 03042020-CSDv | | 34742 | 1403 | 0 |
| | 03042020-CSDv | | 34397 | 1364 | 0 |
| | | **Average particles** | **34776** | **1390** | **0** |
| | | **% Filter collection efficiency** | **87.54%** | **98.63%** | **100.00%** |
| **Sample 13** | 03042020-CSDe | Sample 13,Triple layer Halyard QuickCheck H300 | 22612 | 764 | 1 |
| | 03042020-CSDe | *Background Measurements #1 used for calculation of filter efficiency* | 22557 | 764 | 0 |
| | 03042020-CSDe | | 22314 | 695 | 0 |
| | 03042020-CSDe | | 22681 | 707 | 0 |
| | | **Average particles** | **22541** | **733** | **0** |
| | | **% Filter collection efficiency** | **94.10%** | **99.51%** | **99.98%** |

| | | | | | |
|---|---|---|---|---|---|
| **Sample 14** | 03042020-CSDf | Sample 14,Triple layer Halyard QuickCheck H300 | 24783 | 961 | 0 |
| | 03042020-CSDf | *Background Measurements #1 used for calculation of filter efficiency* | 24535 | 863 | 0 |
| | 03042020-CSDf | | 24631 | 894 | 0 |
| | 03042020-CSDf | | 24110 | 907 | 0 |
| | | **Average particles** | **24515** | **906** | **0** |
| | | **% Filter collection efficiency** | **93.58%** | **99.39%** | **100.00%** |
| **Sample 15** | 03042020-CSDi | Sample 15, Single layer Halyard QuickCheck H300 | 128766 | 19050 | 4 |
| | 03042020-CSDi | Sterilized: 1x, 121 degrees Celsius, 2 atm, 15 min | 128336 | 19107 | 11 |
| | 03042020-CSDi | *Background Measurements #1 used for calculation of filter efficiency* | 130178 | 19365 | 8 |
| | 03042020-CSDi | | 129979 | 19384 | 6 |
| | | **Average particles** | **129315** | **19227** | **7** |
| | | **% Filter collection efficiency** | **66.15%** | **87.14%** | **99.40%** |
| **Sample 16** | 03042020-CSDj | Sample 16, Single layer Halyard QuickCheck H300 | 127472 | 19303 | 2 |
| | 03042020-CSDj | Sterilized: 1x, 121 degrees Celsius, 2 atm, 15 min | 127351 | 18840 | 2 |
| | 03042020-CSDj | *Background Measurements #1 used for calculation of filter efficiency* | 128150 | 19062 | 6 |
| | 03042020-CSDj | | 127637 | 19087 | 3 |
| | | **Average particles** | **127653** | **19073** | **3** |
| | | **% Filter collection efficiency** | **66.58%** | **87.24%** | **99.73%** |
| **Sample 17** | 03042020-CSDg | Double layer Halyard QuickCheck H300 | 73850 | 6206 | 0 |
| | 03042020-CSDg | Sterilized: 1x, 121 degrees Celsius, 2 atm, 15 min | 74493 | 6231 | 0 |
| | 03042020-CSDg | *Background Measurements #1 used for calculation of filter efficiency* | 73894 | 6121 | 0 |
| | 03042020-CSDg | | 73775 | 5892 | 0 |
| | | **Average particles** | **74003** | **6113** | **0** |
| | | **% Filter collection efficiency** | **80.63%** | **95.91%** | **100.00%** |
| **Sample 18** | 03042020-CSDh | Triple layer Halyard QuickCheck H300 | 75495 | 6477 | 0 |
| | 03042020-CSDh | Sterilized: 1x, 121 degrees Celsius, 2 atm, 15 min | 76244 | 6537 | 0 |
| | 03042020-CSDh | *Background Measurements #1 used for calculation of filter efficiency* | 75886 | 6565 | 1 |
| | 03042020-CSDh | | 75530 | 6291 | 0 |
| | | **Average particles** | **75789** | **6468** | **0** |
| | | **% Filter collection efficiency** | **80.16%** | **95.67%** | **99.98%** |

| | | | | | |
|---|---|---|---|---|---|
| **Sample 19** | 03042020-CSDk | Triple layer Halyard QuickCheck H300 | 46148 | 2485 | 2 |
| | 03042020-CSDk | Sterilized: 1x, 121 degrees Celsius, 2 atm, 115 min | 45969 | 2544 | 0 |
| | 03042020-CSDk | *Background Measurements #1 used for calculation of filter efficiency* | 46072 | 2350 | 0 |
| | 03042020-CSDk | | 45516 | 2469 | 0 |
| | | **Average particles** | **45926** | **2462** | **1** |
| | | **% Filter collection efficiency** | **87.98%** | **98.35%** | **99.96%** |

| | | | | | |
|---|---|---|---|---|---|
| **Sample 20** | 03042020-CSDl | Triple layer Halyard QuickCheck H300 | 43836 | 2260 | 0 |
| | 03042020-CSDl | Sterilized: 1x, 121 degrees Celsius, 2 atm, 15 min | 43722 | 2211 | 1 |
| | 03042020-CSDl | *Background Measurements #1 used for calculation of filter efficiency* | 43386 | 2267 | 0 |
| | 03042020-CSDl | | 43664 | 2133 | 0 |
| | | **Average particles** | **43652** | **2218** | **0** |
| | | **% Filter collection efficiency** | **88.57%** | **98.52%** | **99.98%** |

| | | | |
|---|---|---|---|
| **Background measurements #2** | 281752 | 101437 | 635 |
| | 281570 | 102470 | 655 |
| | 277974 | 101341 | 705 |
| | 275088 | 100446 | 629 |
| **Average particles** | **279096** | **101424** | **656** |

| | | | | | |
|---|---|---|---|---|---|
| **Sample 53** | 03042020-CSDp | Double layer Halyard QuickCheck H300 | 43671 | 2462 | 0 |
| | 03042020-CSDp | *Background Measurements #2 used for calculation of filter efficiency* | 43601 | 2650 | 0 |
| | 03042020-CSDp | | 43111 | 2504 | 0 |
| | 03042020-CSDp | | 43531 | 2541 | 1 |
| | | **Average particles** | **43479** | **2539** | **0** |
| | | **% Filter collection efficiency** | **84.42%** | **97.50%** | **99.96%** |

| | | | | | |
|---|---|---|---|---|---|
| **Background Measurements #3** | 12-05-2020 11:19:18 | | 199179 | 53458 | 3000 |
| | 12-05-2020 11:20:18 | | 200037 | 54147 | 3313 |
| | 12-05-2020 11:21:18 | | 197918 | 52847 | 2976 |
| | 12-05-2020 11:22:18 | | 197892 | 52014 | 3034 |
| | | **Average** | **198757** | **53117** | **3081** |
| **Sample 21** | 12-05-2020 11:24:06 | Triple layer Halyard QuickCheck H300 | 34132 | 582 | 4 |
| | 12-05-2020 11:25:06 | Sterilized: 1x, 121 degrees Celsius, 2 atm, 15 min | 34335 | 537 | 5 |
| | 12-05-2020 11:26:06 | *Background Measurements #3 used for calculation of filter efficiency* | 34000 | 587 | 1 |
| | 12-05-2020 11:27:06 | | 34727 | 555 | 1 |
| | | **Average** | **34299** | **565** | **3** |
| | | **% Filter collection efficiency** | **82.74%** | **98.94%** | **99.91%** |
| **Sample 22** | 12-05-2020 11:29:04 | Triple layer Halyard QuickCheck H300 | 32473 | 540 | 4 |
| | 12-05-2020 11:30:04 | Sterilized: 1x, 121 degrees Celsius, 2 atm, 15 min | 32532 | 518 | 0 |
| | 12-05-2020 11:31:04 | *Background Measurements #3 used for calculation of filter efficiency* | 32269 | 513 | 0 |
| | 12-05-2020 11:32:04 | | 32237 | 519 | 0 |
| | | **Average** | **32378** | **523** | **1** |
| | | **% Filter collection efficiency** | **83.71%** | **99.02%** | **99.97%** |
| **Sample 23** | 12-05-2020 11:33:56 | Triple layer Halyard QuickCheck H300 | 32294 | 552 | 0 |
| | 12-05-2020 11:34:56 | Sterilized: 1x, 121 degrees Celsius, 2 atm, 15 min | 32251 | 539 | 1 |
| | 12-05-2020 11:35:56 | *Background Measurements #3 used for calculation of filter efficiency* | 32426 | 545 | 0 |
| | 12-05-2020 11:36:56 | | 32066 | 523 | 0 |
| | | **Average** | **32259** | **540** | **0** |
| | | **% Filter collection efficiency** | **83.77%** | **98.98%** | **99.99%** |
| **Sample 24** | 12-05-2020 11:38:54 | Triple layer Halyard QuickCheck H300 | 32096 | 533 | 3 |
| | 12-05-2020 11:39:54 | Sterilized: 1x, 121 degrees Celsius, 2 atm, 15 min | 32039 | 577 | 0 |
| | 12-05-2020 11:40:54 | *Background Measurements #3 used for calculation of filter efficiency* | 32080 | 554 | 0 |
| | 12-05-2020 11:41:54 | | 31669 | 571 | 1 |
| | | **Average** | **31971** | **559** | **1** |
| | | **% Filter efficiency** | **83.91%** | **98.95%** | **99.97%** |

| | | | | | |
|---|---|---|---|---|---|
| **Background Measurements #4** | | 12-05-2020 11:43:19 | 199586 | 54589 | 3010 |
| | | 12-05-2020 11:44:19 | 201038 | 54950 | 3182 |
| | | 12-05-2020 11:45:19 | 203365 | 57330 | 3693 |
| | | 12-05-2020 11:46:19 | 203026 | 57191 | 3614 |
| | | **Average particles** | **201754** | **56015** | **3375** |
| **Sample 25** | 12-05-2020 11:48:12 | Triple layer Halyard QuickCheck H300 | 22113 | 285 | 1 |
| | 12-05-2020 11:49:12 | Sterilized: 2x, 121 degrees Celsius, 2 atm, 15 min | 22009 | 247 | 1 |
| | 12-05-2020 11:50:12 | *Background Measurements #4 used for calculation of filter efficiency* | 21980 | 280 | 1 |
| | 12-05-2020 11:51:12 | | 22038 | 259 | 1 |
| | | **Average particles** | **22035** | **268** | **1** |
| | | **% Filter collection efficiency** | **89.08%** | **99.52%** | **99.97%** |
| **Sample 26** | 12-05-2020 11:52:52 | Triple layer Halyard QuickCheck H300 | 27234 | 397 | 0 |
| | 12-05-2020 11:53:52 | Sterilized: 2x, 121 degrees Celsius, 2 atm, 15 min | 26938 | 388 | 4 |
| | 12-05-2020 11:54:52 | *Background Measurements #4 used for calculation of filter efficiency* | 27226 | 451 | 3 |
| | 12-05-2020 11:55:52 | | 27383 | 429 | 2 |
| | | **Average particles** | **27195** | **416** | **2** |
| | | **% Filter collection efficiency** | **86.52%** | **99.26%** | **99.93%** |
| **Sample 27** | 12-05-2020 11:57:29 | Triple layer Halyard QuickCheck H300 | 24091 | 286 | 2 |
| | 12-05-2020 11:58:29 | Sterilized: 2x, 121 degrees Celsius, 2 atm, 15 min | 24304 | 309 | 1 |
| | 12-05-2020 11:59:29 | *Background Measurements #4 used for calculation of filter efficiency* | 24523 | 363 | 1 |
| | 12-05-2020 12:00:29 | | 24132 | 344 | 0 |
| | | **Average particles** | **24263** | **326** | **1** |
| | | **% Filter collection efficiency** | **87.97%** | **99.42%** | **99.97%** |
| **Sample 28** | 12-05-2020 12:02:07 | Triple layer Halyard QuickCheck H300 | 27458 | 393 | 3 |
| | 12-05-2020 12:03:07 | Sterilized: 2x, 121 degrees Celsius, 2 atm, 15 min | 26972 | 377 | 2 |
| | 12-05-2020 12:04:07 | *Background Measurements #4 used for calculation of filter efficiency* | 27180 | 369 | 0 |
| | 12-05-2020 12:05:07 | | 27284 | 409 | 0 |
| | | **Average particles** | **27224** | **387** | **1** |
| | | **% Filter collection efficiency** | **86.51%** | **99.31%** | **99.96%** |

| | | | | | |
|---|---|---|---|---|---|
| **Background Measurements #5** | | 12-05-2020 12:06:30 | 203253 | 59450 | 3682 |
| | | 12-05-2020 12:07:30 | 203574 | 60303 | 4160 |
| | | 12-05-2020 12:08:30 | 202157 | 59967 | 4002 |
| | | 12-05-2020 12:09:30 | 203039 | 60223 | 3970 |
| | | **Average particles** | **203006** | **59986** | **3954** |

| | | | | | |
|---|---|---|---|---|---|
| **Sample 29** | 12-05-2020 12:10:58 | Triple layer Halyard QuickCheck H300 | 36750 | 774 | 2 |
| | 12-05-2020 12:11:58 | Sterilized: 3x, 121 degrees Celsius, 2 atm, 15 min | 37271 | 743 | 2 |
| | 12-05-2020 12:12:58 | *Background Measurements #5 used for calculation of filter efficiency* | 37511 | 752 | 0 |
| | 12-05-2020 12:13:58 | | 36993 | 748 | 0 |
| | | **Average particles** | **37131** | **754** | **1** |
| | | **% Filter collection efficiency** | **81.71%** | **98.74%** | **99.97%** |

| | | | | | |
|---|---|---|---|---|---|
| **Sample 30** | 12-05-2020 12:15:43 | Triple layer Halyard QuickCheck H300 | 34559 | 682 | 11 |
| | 12-05-2020 12:16:43 | Sterilized: 3x, 121 degrees Celsius, 2 atm, 15 min | 34648 | 596 | 0 |
| | 12-05-2020 12:17:43 | *Background Measurements #5 used for calculation of filter efficiency* | 34888 | 634 | 2 |
| | 12-05-2020 12:18:43 | | 34920 | 629 | 0 |
| | | **Average particles** | **34754** | **635** | **3** |
| | | **% Filter collection efficiency** | **82.88%** | **98.94%** | **99.92%** |

| | | | | | |
|---|---|---|---|---|---|
| **Sample 31** | 12-05-2020 12:20:37 | Triple layer Halyard QuickCheck H300 | 33308 | 604 | 1 |
| | 12-05-2020 12:21:37 | Sterilized: 3x, 121 degrees Celsius, 2 atm, 15 min | 33013 | 560 | 1 |
| | 12-05-2020 12:22:37 | *Background Measurements #5 used for calculation of filter efficiency* | 33233 | 605 | 2 |
| | 12-05-2020 12:23:37 | | 33137 | 618 | 0 |
| | | **Average particles** | **33173** | **597** | **1** |
| | | **% Filter collection efficiency** | **83.66%** | **99.01%** | **99.97%** |

| | | | | | |
|---|---|---|---|---|---|
| **Sample 32** | 12-05-2020 12:25:10 | Triple layer Halyard QuickCheck H300 | 36298 | 683 | 0 |
| | 12-05-2020 12:26:10 | Sterilized: 3x, 121 degrees Celsius, 2 atm, 15 min | 36499 | 707 | 0 |
| | 12-05-2020 12:27:10 | *Background Measurements #5 used for calculation of filter efficiency* | 36445 | 699 | 1 |
| | 12-05-2020 12:28:10 | | 36351 | 788 | 0 |
| | | **Average particles** | **36398** | **719** | **0** |
| | | **% Filter collection efficiency** | **82.07%** | **98.80%** | **99.99%** |

| | | | | | |
|---|---|---|---|---|---|
| **Background Measurements #6** | | 12-05-2020 12:29:33 | 201424 | 60674 | 4051 |
| | | 12-05-2020 12:30:33 | 201432 | 60566 | 4109 |
| | | 12-05-2020 12:31:33 | 202001 | 60854 | 4089 |
| | | 12-05-2020 12:32:33 | 198776 | 59439 | 3744 |
| | | **Average particles** | **200908** | **60383** | **3998** |

| | | | | | |
|---|---|---|---|---|---|
| **Sample 33** | 12-05-2020 12:34:01 | Triple layer Halyard QuickCheck H300 | 28241 | 461 | 3 |
| | 12-05-2020 12:35:01 | Sterilized: 4x, 121 degrees Celsius, 2 atm, 15 min | 28206 | 423 | 0 |
| | 12-05-2020 12:36:01 | *Background Measurements #6 used for calculation of filter efficiency* | 28117 | 445 | 3 |
| | 12-05-2020 12:37:01 | | 27944 | 445 | 1 |
| | | **Average particles** | **28127** | **444** | **2** |
| | | **% Filter collection efficiency** | **86.00%** | **99.27%** | **99.96%** |

| | | | | | |
|---|---|---|---|---|---|
| **Sample 34** | 12-05-2020 12:38:38 | Triple layer Halyard QuickCheck H300 | 27216 | 408 | 2 |
| | 12-05-2020 12:39:38 | Sterilized: 4x, 121 degrees Celsius, 2 atm, 15 min | 27672 | 469 | 9 |
| | 12-05-2020 12:40:38 | *Background Measurements #6 used for calculation of filter efficiency* | 27168 | 417 | 0 |
| | 12-05-2020 12:41:38 | | 27544 | 429 | 5 |
| | | **Average particles** | **27400** | **431** | **4** |
| | | **% Filter collection efficiency** | **86.36%** | **99.29%** | **99.90%** |

| | | | | | |
|---|---|---|---|---|---|
| **Sample 35** | 12-05-2020 12:43:11 | Triple layer Halyard QuickCheck H300 | 29020 | 487 | 2 |
| | 12-05-2020 12:44:11 | Sterilized: 4x, 121 degrees Celsius, 2 atm, 15 min | 29331 | 483 | 3 |
| | 12-05-2020 12:45:11 | *Background Measurements #6 used for calculation of filter efficiency* | 28752 | 470 | 0 |
| | 12-05-2020 12:46:11 | | 28601 | 463 | 0 |
| | | **Average particles** | **28926** | **476** | **1** |
| | | **% Filter collection efficiency** | **85.60%** | **99.21%** | **99.97%** |

| | | | | | |
|---|---|---|---|---|---|
| **Sample 36** | 12-05-2020 12:47:49 | Triple layer Halyard QuickCheck H300 | 29645 | 521 | 10 |
| | 12-05-2020 12:48:49 | Sterilized: 4x, 121 degrees Celsius, 2 atm, 15 min | 29461 | 496 | 0 |
| | 12-05-2020 12:49:49 | *Background Measurements #6 used for calculation of filter efficiency* | 29765 | 514 | 1 |
| | 12-05-2020 12:50:49 | | 29472 | 484 | 1 |
| | | **Average particles** | **29586** | **504** | **3** |
| | | **% Filter collection efficiency** | **85.27%** | **99.17%** | **99.92%** |

| | | | | | |
|---|---|---|---|---|---|
| **Background Measurements #7** | | 12-05-2020 12:52:20 | 193547 | 57697 | 3775 |
| | | 12-05-2020 12:53:20 | 195559 | 58490 | 3808 |
| | | 12-05-2020 12:54:20 | 195140 | 58454 | 3796 |
| | | 12-05-2020 12:55:20 | 195848 | 59217 | 3953 |
| | | **Average particles** | **195024** | **58465** | **3833** |
| **Sample 37** | 12-05-2020 12:56:49 | Triple layer Halyard QuickCheck H300 | 31897 | 582 | 2 |
| | 12-05-2020 12:57:49 | Sterilized: 5x, 121 degrees Celsius, 2 atm, 15 min | 32117 | 623 | 3 |
| | 12-05-2020 12:58:49 | *Background Measurements #7 used for calculation of filter efficiency* | 32102 | 589 | 2 |
| | 12-05-2020 12:59:49 | | 31754 | 553 | 0 |
| | | **Average particles** | **31968** | **587** | **2** |
| | | **% Filter collection efficiency** | **83.61%** | **99.00%** | **99.95%** |
| **Sample 38** | 12-05-2020 13:01:21 | Triple layer Halyard QuickCheck H300 | 29977 | 494 | 0 |
| | 12-05-2020 13:02:21 | Sterilized: 5x, 121 degrees Celsius, 2 atm, 15 min | 29600 | 460 | 0 |
| | 12-05-2020 13:03:21 | *Background Measurements #7 used for calculation of filter efficiency* | 29845 | 543 | 2 |
| | 12-05-2020 13:04:21 | | 29843 | 474 | 1 |
| | | **Average particles** | **29816** | **493** | **1** |
| | | **% Filter collection efficiency** | **84.71%** | **99.16%** | **99.98%** |
| **Sample 39** | 12-05-2020 13:05:56 | Triple layer Halyard QuickCheck H300 | 32389 | 559 | 4 |
| | 12-05-2020 13:06:56 | Sterilized: 5x, 121 degrees Celsius, 2 atm, 15 min | 32570 | 620 | 0 |
| | 12-05-2020 13:07:56 | *Background Measurements #7 used for calculation of filter efficiency* | 33131 | 643 | 1 |
| | 12-05-2020 13:08:56 | | 33075 | 572 | 1 |
| | | **Average particles** | **32791** | **599** | **2** |
| | | **% Filter collection efficiency** | **83.19%** | **98.98%** | **99.96%** |
| **Sample 40** | 12-05-2020 13:10:35 | Triple layer Halyard QuickCheck H300 | 34261 | 659 | 0 |
| | 12-05-2020 13:11:35 | Sterilized: 5x, 121 degrees Celsius, 2 atm, 15 min | 34125 | 650 | 0 |
| | 12-05-2020 13:12:35 | *Background Measurements #7 used for calculation of filter efficiency* | 34417 | 627 | 1 |
| | 12-05-2020 13:13:35 | | 34357 | 680 | 1 |
| | | **Average particles** | **34290** | **654** | **1** |
| | | **% Filter collection efficiency** | **82.42%** | **98.88%** | **99.99%** |

| | | | | |
|---|---|---|---|---|
| **Background Measurements #8** | | **234853** | **36280** | **1157** |
| **Sample 41** | Surgical Mask | 103596 | 3897 | 12 |
| **Sample 42** | *Background Measurement #8 used for calculation of filter efficiency* | 113947 | 4545 | 23 |
| **Sample 43** | | 99473 | 3680 | 6 |
| **Sample 44** | | 110228 | 4412 | 9 |
| | **Average** | **106811** | **4133.5** | **12.5** |
| | **Filter efficiency** | **54.52%** | **88.61%** | **98.92%** |
| | **Filter efficiency Std. Dev.** | **2.77%** | **1.13%** | **0.64%** |
| **Sample 45** | Disposable Face Mask FFP2 | 15138 | 175 | 0 |
| **Sample 46** | *Background Measurement #8 used for calculation of filter efficiency* | 14251 | 154 | 0 |
| **Sample 47** | | 13265 | 142 | 0 |
| **Sample 48** | | 12986 | 155 | 0 |
| | **Average** | **13910** | **156.5** | **0** |
| | **Filter efficiency** | **94.08%** | **99.57%** | **100.00%** |
| | **Filter efficiency Std. Dev.** | **0.42%** | **0.04%** | **0.00%** |
| **Sample 49** | 3M 8320 FFP2 NR D Mask | 2550 | 35 | 1 |
| **Sample 50** | *Background Measurement #8 used for calculation of filter efficiency* | 9606 | 128 | 0 |
| **Sample 51** | | 4465 | 79 | 0 |
| **Sample 52** | | 7419 | 127 | 2 |
| | **Average** | **6010** | **92.25** | **0.75** |
| | **Filter efficiency** | **97.44%** | **99.75%** | **99.94%** |
| | **Filter efficiency Std. Dev.** | **1.33%** | **0.12%** | **0.08%** |
