# Supplementary material for: Using surgical wrapping material for the fabrication of respirator masks
Source: PLoS One. 2020 Jul 21;15(7):e0236239. doi: 10.1371/journal.pone.0236239 (PMC7373262; doi:10.1371/journal.pone.0236239)
Supplement: S3 Data — (PDF) [file pone.0236239.s003.pdf]

Differential Pressure test

				cmH2O	Pa	Pa/cm2		
**Unsterilised material (from package)**	White Layer	Sample P1	20/5/2020	2.3	225.6	25.63		
		Sample P2	20/5/2020	2.1	205.9	23.40		
		Sample P3	20/5/2020	1.9	186.3	21.17	***μ***	***σ***
		Sample P4	20/5/2020	2.4	235.4	26.75	24.2	2.5
	Blue Layer	Sample P5	20/5/2020	2.8	274.6	31.20		
		Sample P6	20/5/2020	3	294.2	33.43		
		Sample P7	20/5/2020	2.6	255.0	28.97	***μ***	***σ***
		Sample P8	20/5/2020	2.8	274.6	31.20	31.2	1.8
	Single layer	Sample P9	20/5/2020	5.2	509.9	57.95		
		Sample P10	20/5/2020	4.7	460.9	52.38		
		Sample P11	20/5/2020	4.6	451.1	51.26	***μ***	***σ***
		Sample P12	20/5/2020	5.1	500.1	56.83	54.6	3.3
	Double layer	Sample P13	20/5/2020	10.8	1059.1	120.35		
		Sample P14	20/5/2020	10.1	990.5	112.55		
		Sample P15	20/5/2020	10.1	990.5	112.55	***μ***	***σ***
		Sample P16	20/5/2020	12.2	1196.4	135.96	120.4	11.0
	Triple layer	Sample P17	20/5/2020	14.6	1431.8	162.70		
		Sample P18	20/5/2020	16.4	1608.3	182.76		
		Sample P19	20/5/2020	15.9	1559.3	177.19	***μ***	***σ***
		Sample P20	20/5/2020	16.6	1627.9	184.99	176.9	10.0
**Steam Sterilised (15min, 121 degrees Celsius, 2 atm)**	Triple layer, 1x sterilized	Sample P21	20/5/2020	18	1765.2	200.59		
		Sample P22	20/5/2020	16.6	1627.9	184.99		
		Sample P23	20/5/2020	18.5	1814.2	206.16	***μ***	***σ***

		Sample P24	20/5/2020	16.1	1578.9	179.42	192.8	12.6
	Triple layer, 2x sterilized	Sample P25	20/5/2020	17.5	1716.2	195.02		
		Sample P26	20/5/2020	15.2	1490.6	169.39		
		Sample P27	20/5/2020	16.4	1608.3	182.76	***μ***	***σ***
		Sample P28	20/5/2020	16.1	1578.9	179.42	181.6	10.6
	Triple layer, 3x sterilized	Sample P29	20/5/2020	15.9	1559.3	177.19		
		Sample P30	20/5/2020	16.6	1627.9	184.99		
		Sample P31	20/5/2020	16.3	1598.5	181.65	***μ***	***σ***
		Sample P32	20/5/2020	16.9	1657.3	188.33	183.0	4.8
	Triple layer, 4x sterilized	Sample P33	20/5/2020	15.7	1539.6	174.96		
		Sample P34	20/5/2020	16.1	1578.9	179.42		
		Sample P35	20/5/2020	16.2	1588.7	180.53	***μ***	***σ***
		Sample P36	20/5/2020	15.6	1529.8	173.85	177.2	3.3
	Triple layer, 5x sterilized	Sample P37	20/5/2020	14.7	1441.6	163.82		
		Sample P38	20/5/2020	16.2	1588.7	180.53		
		Sample P39	20/5/2020	15.6	1529.8	173.85	***μ***	***σ***
		Sample P40	20/5/2020	15.7	1539.6	174.96	173.3	7.0
**Comparison with commercial**	Surgical Mask	Sample P41	20/5/2020	3.7	362.8	41.23		
**Masks & Respirators**	Ghor-1105-3	Sample P42	20/5/2020	3.3	323.6	36.77		
	Henan Gore Medical Instrume	Sample P43	20/5/2020	3.5	343.2	39.00	***μ***	***σ***
		Sample P44	20/5/2020	3.2	313.8	35.66	38.2	2.5
	Disposable Face Mask FFP2	Sample P45	20/5/2020	8.6	843.4	95.84		
	EN149:2001+A1: 2009	Sample P46	20/5/2020	8.8	863.0	98.07		
	GHOR-1105-5	Sample P47	20/5/2020	8.9	872.8	99.18	***μ***	***σ***
		Sample P48	20/5/2020	9.1	892.4	101.41	98.6	2.3

3M 8320 FFP2 NR D	Sample P49	20/5/2020	6.6	647.2	73.55		
	Sample P50	20/5/2020	6.2	608.0	69.09		
	Sample P51	20/5/2020	5.2	509.9	57.95	***μ***	***σ***
	Sample P52	20/5/2020	5.6	549.2	62.41	65.7	6.9
