# Supplementary material for: Using surgical wrapping material for the fabrication of respirator masks
Source: PLoS One. 2020 Jul 21;15(7):e0236239. doi: 10.1371/journal.pone.0236239 (PMC7373262; doi:10.1371/journal.pone.0236239)
Supplement: S4 Data — (PDF) [file pone.0236239.s004.pdf]

Water column pressure test

Unsterilised material (from package)

			cm H20		
White Layer only	Sample PT52	20/05/2020	55		
	Sample PT53	20/05/2020	49		
	Sample PT54	20/05/2020	52	***μ***	***σ***
	Sample PT55	20/05/2020	46	50.5	3.9
Blue layer only	Sample PT56	20/05/2020	86		
	Sample PT57	20/05/2020	92		
	Sample PT58	20/05/2020	82	***μ***	***σ***
	Sample PT59	20/05/2020	81	85.3	5.0
Single Layer	Sample PT1	3/4/2020	95		
	Sample PT2	3/4/2020	85		
	Sample PT3	3/4/2020	85	***μ***	***σ***
	Sample PT60	20/05/2020	79	86.0	6.6
Double Layer	Sample PT4	3/4/2020	95		
	Sample PT5	3/4/2020	90		
	Sample PT6	3/4/2020	90	***μ***	***σ***
	Sample PT61	20/05/2020	94	92.3	2.6
Double Layer, Reversed	Sample PT62	20/05/2020	92		
	Sample PT63	20/05/2020	92		
	Sample PT64	20/05/2020	94	***μ***	***σ***
	Sample PT65	20/05/2020	96	93.5	1.9
Triple Layer	Sample PT7	3/4/2020	95		
	Sample PT8	3/4/2020	115		
	Sample PT9	3/4/2020	105		

		Sample PT16	12/5/2020	102		
		Sample PT17	12/5/2020	93		
		Sample PT18	12/5/2020	94		
		Sample PT19	12/5/2020	90		
		Sample PT66	20/05/2020	91		
		Sample PT67	20/05/2020	106		
		Sample PT68	20/05/2020	94	***μ***	***σ***
		Sample PT69	20/05/2020	96	98.3	7.7
**Steam Sterilised (15min, 121 degrees Celsius, 2 atm)**	Single Layer, 1x Sterilized	Sample PT10	3/4/2020	80		
		Sample PT11	3/4/2020	80		
		Sample PT70	20/05/2020	77	***μ***	***σ***
		Sample PT71	20/05/2020	81	79.5	1.7
	Double Layer, 1x Sterilized	Sample PT12	3/4/2020	90		
		Sample PT13	3/4/2020	90		
		Sample PT72	20/05/2020	88	***μ***	***σ***
		Sample PT73	20/05/2020	87	88.8	1.5
	Triple Layer, 1x Sterilized	Sample PT14	3/4/2020	100		
		Sample PT15	3/4/2020	100		
		Sample PT20	12/5/2020	98		
		Sample PT21	12/5/2020	93		
		Sample PT22	12/5/2020	94	***μ***	***σ***
		Sample PT23	12/5/2020	92	96.2	3.6
	Triple Layer, 2x Sterilized	Sample PT24	12/5/2020	101		
		Sample PT25	12/5/2020	99		
		Sample PT26	12/5/2020	104	***μ***	***σ***
		Sample PT27	12/5/2020	99	100.8	2.4

Triple Layer, 3x Sterilized	Sample PT28	12/5/2020	90		
	Sample PT29	12/5/2020	95		
	Sample PT30	12/5/2020	93	***μ***	***σ***
	Sample PT31	12/5/2020	92	92.5	2.1

Triple Layer, 4x Sterilized	Sample PT32	12/5/2020	99		
	Sample PT33	12/5/2020	103		
	Sample PT34	12/5/2020	106	***μ***	***σ***
	Sample PT35	12/5/2020	104	103.0	2.9

Triple Layer, 5x Sterilized	Sample PT36	12/5/2020	94		
	Sample PT37	12/5/2020	97		
	Sample PT38	12/5/2020	95	***μ***	***σ***
	Sample PT39	12/5/2020	93	94.8	1.7

Comparison with commercial Masks & Respirators

Surgical Mask	Sample PT40	20/05/2020	62		
	Sample PT41	20/05/2020	67		
	Sample PT42	20/05/2020	64	***μ***	***σ***
	Sample PT43	20/05/2020	69	65.5	3.1

Disposable Face Mask FFP2	Sample PT44	20/05/2020	96		
	Sample PT45	20/05/2020	89		
	Sample PT46	20/05/2020	92	***μ***	***σ***
	Sample PT47	20/05/2020	93	92.5	2.9

3M 8320 FFP2 NR D	Sample PT48	20/05/2020	56		
	Sample PT49	20/05/2020	61		
	Sample PT50	20/05/2020	53	***μ***	***σ***
	Sample PT51	20/05/2020	51	55.3	4.3
