# Supplementary material for: Using surgical wrapping material for the fabrication of respirator masks
Source: PLoS One. 2020 Jul 21;15(7):e0236239. doi: 10.1371/journal.pone.0236239 (PMC7373262; doi:10.1371/journal.pone.0236239)
Supplement: S5 Data — (PDF) [file pone.0236239.s005.pdf]

# Filter Efficiency Test H500

| | | | 0.3um | 0.5um | 3.0um |
|---|---|---|---|---|---|
| **White Layer Only, H500** | Sample H500-13 | 28/05/2020 | 71.46% | 91.02% | 99.47% |
| | Sample H500-14 | 28/05/2020 | 67.20% | 88.31% | 99.19% |
| | Sample H500-15 | 28/05/2020 | 67.16% | 88.19% | 98.87% |
| | Sample H500-16 | 28/05/2020 | 74.31% | 92.88% | 99.41% |
| | | **Averaged** | **70.03%** | **90.10%** | **99.23%** |
| | | **Intersample Standard Dev.** | **3.49%** | **2.27%** | **0.27%** |
| **Blue layer Only, H500** | Sample H500-17 | 28/05/2020 | 69.12% | 89.05% | 99.20% |
| | Sample H500-18 | 28/05/2020 | 71.02% | 90.43% | 99.40% |
| | Sample H500-19 | 28/05/2020 | 70.16% | 89.53% | 99.45% |
| | Sample H500-20 | 28/05/2020 | 70.71% | 89.72% | 99.32% |
| | | **Averaged** | **70.25%** | **89.68%** | **99.34%** |
| | | **Intersample Standard Dev.** | **0.84%** | **0.57%** | **0.11%** |
| **Single layer, H500** | Sample H500-1 | 28/05/2020 | 87.63% | 98.33% | 99.89% |
| | Sample H500-2 | 28/05/2020 | 86.40% | 97.95% | 99.96% |
| | Sample H500-3 | 28/05/2020 | 85.67% | 97.61% | 99.98% |
| | Sample H500-4 | 28/05/2020 | 86.86% | 98.01% | 99.96% |
| | | **Averaged** | **86.64%** | **97.98%** | **99.95%** |
| | | **Intersample Standard Dev.** | **0.82%** | **0.30%** | **0.04%** |
| **Double Layer, H500** | Sample H500-5 | 28/05/2020 | 97.08% | 99.86% | 99.97% |
| | Sample H500-6 | 28/05/2020 | 96.00% | 99.76% | 99.97% |
| | Sample H500-7 | 28/05/2020 | 96.48% | 99.79% | 99.97% |
| | Sample H500-8 | 28/05/2020 | 97.46% | 99.88% | 99.98% |
| | | **Averaged** | **96.75%** | **99.82%** | **99.97%** |
| | | **Intersample Standard Dev.** | **0.65%** | **0.06%** | **0.01%** |

| | | | | | |
|---|---|---|---|---|---|
| **Triple Layer, H500** | Sample H500-9 | 28/05/2020 | 99.22% | 99.92% | 99.75% |
| | Sample H500-10 | 28/05/2020 | 99.01% | 99.97% | 99.99% |
| | Sample H500-11 | 28/05/2020 | 99.31% | 99.98% | 99.99% |
| | Sample H500-12 | 28/05/2020 | 99.14% | 99.97% | 99.98% |
| | | **Averaged** | **99.17%** | **99.96%** | **99.93%** |
| | | **Intersample Standard Dev.** | **0.13%** | **0.03%** | **0.12%** |

## Filter Efficiency Test H500 (per sample)

| | | | 0,3µm | 0,5µm | 3,0µm |
|---|---|---|---|---|---|
| **Single Layer, H500** | Background measurement | 28-05-2020 16:26:37 | 233933 | 86142 | 1945 |
| | | 28-05-2020 16:27:37 | 227428 | 81748 | 1407 |
| | | 28-05-2020 16:28:37 | 226702 | 82269 | 1277 |
| | | 28-05-2020 16:29:37 | 226390 | 81695 | 1545 |
| | | 28-05-2020 16:41:34 | 222989 | 81879 | 1333 |
| | | 28-05-2020 16:42:34 | 223266 | 82389 | 1316 |
| | | 28-05-2020 16:43:34 | 223463 | 82193 | 1257 |
| | | 28-05-2020 16:44:34 | 221485 | 81769 | 1240 |
| | | **Average particles** | **225707** | **82511** | **1415** |
| | Sample H500-1 | 28-05-2020 16:47:15 | 27852 | 1335 | 0 |
| | | 28-05-2020 16:48:15 | 28133 | 1409 | 3 |
| | | 28-05-2020 16:49:15 | 27904 | 1358 | 2 |
| | | 28-05-2020 16:50:15 | 27768 | 1400 | 1 |
| | | **Average particles** | **27914** | **1376** | **2** |
| | | **% Filter collection efficiency** | **87.63%** | **98.33%** | **99.89%** |
| | Sample H500-2 | 28-05-2020 16:51:46 | 30717 | 1731 | 1 |
| | | 28-05-2020 16:52:46 | 31082 | 1699 | 1 |
| | | 28-05-2020 16:53:46 | 30553 | 1647 | 0 |
| | | 28-05-2020 16:54:46 | 30391 | 1676 | 0 |
| | | **Average particles** | **30686** | **1688** | **1** |
| | | **% Filter collection efficiency** | **86.40%** | **97.95%** | **99.96%** |
| | Sample H500-3 | 28-05-2020 16:56:30 | 32489 | 1937 | 0 |
| | | 28-05-2020 16:57:30 | 31733 | 1876 | 0 |
| | | 28-05-2020 16:58:30 | 32685 | 2062 | 1 |
| | | 28-05-2020 16:59:30 | 32478 | 2017 | 0 |

| | | | | | |
|---|---|---|---|---|---|
| | | **Average particles** | **32346** | **1973** | **0** |
| | | **% Filter collection efficiency** | **85.67%** | **97.61%** | **99.98%** |
| | Sample H500-4 | 28-05-2020 17:01:02 | 30015 | 1705 | 1 |
| | | 28-05-2020 17:02:02 | 29961 | 1631 | 1 |
| | | 28-05-2020 17:03:02 | 29302 | 1648 | 0 |
| | | 28-05-2020 17:04:02 | 29320 | 1573 | 0 |
| | | **Average particles** | **29650** | **1639** | **1** |
| | | **% Filter collection efficiency** | **86.86%** | **98.01%** | **99.96%** |
| | Background measurement | 28-05-2020 17:05:52 | 220648 | 85968 | 1622 |
| | | 28-05-2020 17:06:52 | 217750 | 84829 | 1532 |
| | | 28-05-2020 17:07:52 | 217294 | 84608 | 1558 |
| | | 28-05-2020 17:08:52 | 216690 | 85108 | 1732 |
| | | **Average particles** | **218096** | **85128** | **1611** |
| **Double Layer H500** | Sample H500-5 | 28-05-2020 17:10:47 | 6325 | 134 | 0 |
| | | 28-05-2020 17:11:47 | 6286 | 117 | 0 |
| | | 28-05-2020 17:12:47 | 6379 | 93 | 1 |
| | | 28-05-2020 17:13:47 | 6445 | 133 | 1 |
| | | **Average particles** | **6359** | **119** | **1** |
| | | **% Filter collection efficiency** | **97.08%** | **99.86%** | **99.97%** |
| | Sample H500-6 | 28-05-2020 17:16:22 | 8736 | 172 | 1 |
| | | 28-05-2020 17:17:22 | 8742 | 201 | 0 |
| | | 28-05-2020 17:18:22 | 8713 | 242 | 0 |
| | | 28-05-2020 17:19:22 | 8711 | 205 | 1 |
| | | **Average particles** | **8726** | **205** | **1** |
| | | **% Filter collection efficiency** | **96.00%** | **99.76%** | **99.97%** |
| | Sample H500-7 | 28-05-2020 17:21:18 | 7780 | 177 | 0 |
| | | 28-05-2020 17:22:18 | 7871 | 167 | 1 |

| | | | | | |
|---|---|---|---|---|---|
| | | 28-05-2020 17:23:18 | 7500 | 195 | 1 |
| | | 28-05-2020 17:24:18 | 7577 | 171 | 0 |
| | | **Average particles** | **7682** | **178** | **1** |
| | | **% Filter collection efficiency** | **96.48%** | **99.79%** | **99.97%** |
| | Sample H500-8 | 28-05-2020 17:25:55 | 5589 | 104 | 0 |
| | | 28-05-2020 17:26:55 | 5422 | 81 | 0 |
| | | 28-05-2020 17:27:55 | 5651 | 109 | 0 |
| | | 28-05-2020 17:28:55 | 5509 | 130 | 1 |
| | | **Average particles** | **5543** | **106** | **0** |
| | | **% Filter collection efficiency** | **97.46%** | **99.88%** | **99.98%** |
| **Triple layer, H500** | Background measurement | 28-05-2020 17:30:25 | 210249 | 85747 | 2532 |
| | | 28-05-2020 17:31:25 | 209445 | 85859 | 2592 |
| | | 28-05-2020 17:32:25 | 207826 | 85073 | 2736 |
| | | 28-05-2020 17:33:25 | 207924 | 84831 | 2545 |
| | | **Average particles** | **208861** | **85378** | **2601** |
| | Sample H500-9 | 28-05-2020 17:34:59 | 1747 | 176 | 26 |
| | | 28-05-2020 17:35:59 | 1601 | 30 | 0 |
| | | 28-05-2020 17:36:59 | 1659 | 34 | 0 |
| | | 28-05-2020 17:37:59 | 1512 | 31 | 0 |
| | | **Average particles** | **1630** | **68** | **7** |
| | | **% Filter collection efficiency** | **99.22%** | **99.92%** | **99.75%** |
| | Sample H500-10 | 28-05-2020 17:39:33 | 1984 | 38 | 1 |
| | | 28-05-2020 17:40:33 | 2123 | 22 | 0 |
| | | 28-05-2020 17:41:33 | 2098 | 21 | 0 |
| | | 28-05-2020 17:42:33 | 2049 | 30 | 0 |
| | | **Average particles** | **2064** | **28** | **0** |
| | | **% Filter collection efficiency** | **99.01%** | **99.97%** | **99.99%** |

| | | | | | |
|---|---|---|---|---|---|
| | Sample H500-11 | 28-05-2020 17:44:46 | 1407 | 17 | 0 |
| | | 28-05-2020 17:45:46 | 1423 | 17 | 1 |
| | | 28-05-2020 17:46:46 | 1431 | 19 | 0 |
| | | 28-05-2020 17:47:46 | 1489 | 14 | 0 |
| | | **Average particles** | **1438** | **17** | **0** |
| | | **% Filter collection efficiency** | **99.31%** | **99.98%** | **99.99%** |
| | Sample H500-12 | 28-05-2020 17:49:29 | 1861 | 33 | 0 |
| | | 28-05-2020 17:50:29 | 1700 | 34 | 2 |
| | | 28-05-2020 17:51:29 | 1782 | 20 | 0 |
| | | 28-05-2020 17:52:29 | 1808 | 28 | 0 |
| | | **Average particles** | **1788** | **29** | **1** |
| | | **% Filter collection efficiency** | **99.14%** | **99.97%** | **99.98%** |
| **White layer only, H500** | Background measurement | 28-05-2020 17:54:00 | 211665 | 89667 | 3241 |
| | | 28-05-2020 17:55:00 | 209972 | 89847 | 3322 |
| | | 28-05-2020 17:56:00 | 211880 | 90592 | 3647 |
| | | 28-05-2020 17:57:00 | 207552 | 88315 | 3189 |
| | | **Average particles** | **210267** | **89605** | **3350** |
| | Sample H500-13 | 28-05-2020 17:58:34 | 59356 | 7963 | 19 |
| | | 28-05-2020 17:59:34 | 59592 | 7942 | 18 |
| | | 28-05-2020 18:00:34 | 60115 | 7961 | 18 |
| | | 28-05-2020 18:01:34 | 61005 | 8309 | 16 |
| | | **Average particles** | **60017** | **8044** | **18** |
| | | **% Filter collection efficiency** | **71.46%** | **91.02%** | **99.47%** |
| | Sample H500-14 | 28-05-2020 18:03:13 | 68823 | 10287 | 23 |
| | | 28-05-2020 18:04:13 | 68522 | 10340 | 27 |
| | | 28-05-2020 18:05:13 | 68967 | 10422 | 32 |
| | | 28-05-2020 18:06:13 | 69560 | 10846 | 27 |
| | | **Average particles** | **68968** | **10474** | **27** |

| | | | | | |
|---|---|---|---|---|---|
| | | **% Filter collection efficiency** | **67.20%** | **88.31%** | **99.19%** |
| | Sample H500-15 | 28-05-2020 18:07:42 | 69118 | 10730 | 33 |
| | | 28-05-2020 18:08:42 | 69262 | 10496 | 37 |
| | | 28-05-2020 18:09:42 | 69341 | 10653 | 41 |
| | | 28-05-2020 18:10:42 | 68468 | 10452 | 41 |
| | | **Average particles** | **69047** | **10583** | **38** |
| | | **% Filter collection efficiency** | **67.16%** | **88.19%** | **98.87%** |
| | Sample H500-16 | 28-05-2020 18:12:05 | 54065 | 6368 | 22 |
| | | 28-05-2020 18:13:05 | 53970 | 6393 | 20 |
| | | 28-05-2020 18:14:05 | 53982 | 6406 | 26 |
| | | 28-05-2020 18:15:05 | 54042 | 6360 | 11 |
| | | **Average particles** | **54015** | **6382** | **20** |
| | | **% Filter collection efficiency** | **74.31%** | **92.88%** | **99.41%** |
| **Blue Layer only, H500** | Background measurement | 28-05-2020 18:16:29 | 214102 | 93238 | 3588 |
| | | 28-05-2020 18:17:29 | 214604 | 93205 | 3550 |
| | | 28-05-2020 18:18:29 | 213782 | 92229 | 3430 |
| | | 28-05-2020 18:19:29 | 212550 | 91262 | 3281 |
| | | **Average particles** | **213760** | **92484** | **3462** |
| | Sample H500-17 | 28-05-2020 18:35:52 | 66719 | 10039 | 30 |
| | | 28-05-2020 18:36:52 | 65911 | 10091 | 33 |
| | | 28-05-2020 18:37:52 | 65244 | 10096 | 27 |
| | | 28-05-2020 18:38:52 | 66161 | 10294 | 21 |
| | | **Average particles** | **66009** | **10130** | **28** |
| | | **% Filter collection efficiency** | **69.12%** | **89.05%** | **99.20%** |
| | Sample H500-18 | 28-05-2020 18:40:24 | 62043 | 9003 | 24 |
| | | 28-05-2020 18:41:24 | 61942 | 8695 | 21 |
| | | 28-05-2020 18:42:24 | 61879 | 8869 | 17 |

| | | | | |
|---|---|---|---|---|
| | 28-05-2020 18:43:24 | 61894 | 8832 | 21 |
| | **Average particles** | **61940** | **8850** | **21** |
| | **% Filter collection efficiency** | **71.02%** | **90.43%** | **99.40%** |
| Sample H500-19 | 28-05-2020 18:44:56 | 64204 | 9746 | 26 |
| | 28-05-2020 18:45:56 | 63711 | 9603 | 22 |
| | 28-05-2020 18:46:56 | 63514 | 9634 | 10 |
| | 28-05-2020 18:47:56 | 63717 | 9738 | 18 |
| | **Average particles** | **63787** | **9680** | **19** |
| | **% Filter collection efficiency** | **70.16%** | **89.53%** | **99.45%** |
| Sample H500-20 | 28-05-2020 18:49:20 | 62734 | 9460 | 25 |
| | 28-05-2020 18:50:20 | 62818 | 9537 | 20 |
| | 28-05-2020 18:51:20 | 62134 | 9462 | 18 |
| | 28-05-2020 18:52:20 | 62727 | 9585 | 31 |
| | **Average particles** | **62603** | **9511** | **24** |
| | **% Filter collection efficiency** | **70.71%** | **89.72%** | **99.32%** |

## Differential Pressure test H500

**Unsterilised H500 material (from package)**

| | | | cmH2O | Pa | Pa/cm2 | | |
|---|---|---|---|---|---|---|---|
| White Layer | Sample H1 | 28/5/2020 | 6.5 | 637.4 | 72.44 | | |
| | Sample H2 | 28/5/2020 | 5.4 | 529.6 | 60.18 | | |
| | Sample H3 | 28/5/2020 | 5.1 | 500.1 | 56.83 | ***μ*** | ***σ*** |
| | Sample H4 | 28/5/2020 | 4.5 | 441.3 | 50.15 | 59.9 | 9.3 |
| Blue Layer | Sample H5 | 28/5/2020 | 3.7 | 362.8 | 41.23 | | |
| | Sample H6 | 28/5/2020 | 3.5 | 343.2 | 39.00 | | |
| | Sample H7 | 28/5/2020 | 4 | 392.3 | 44.58 | ***μ*** | ***σ*** |
| | Sample H8 | 28/5/2020 | 4 | 392.3 | 44.58 | 42.3 | 2.7 |
| Single layer | Sample H9 | 28/5/2020 | 9.1 | 892.4 | 101.41 | | |
| | Sample H10 | 28/5/2020 | 10.3 | 1010.1 | 114.78 | | |
| | Sample H11 | 28/5/2020 | 9.6 | 941.4 | 106.98 | ***μ*** | ***σ*** |
| | Sample H12 | 28/5/2020 | 10.5 | 1029.7 | 117.01 | 110.0 | 7.2 |
| Double layer | Sample H13 | 28/5/2020 | 20.4 | 2000.6 | 227.34 | | |
| | Sample H14 | 28/5/2020 | 18.9 | 1853.5 | 210.62 | | |
| | Sample H15 | 28/5/2020 | 19 | 1863.3 | 211.73 | ***μ*** | ***σ*** |
| | Sample H16 | 28/5/2020 | 20.2 | 1980.9 | 225.11 | 218.7 | 8.7 |
| Triple layer | Sample H17 | 28/5/2020 | 29 | 2843.9 | 323.17 | | |
| | Sample H18 | 28/5/2020 | 32.9 | 3226.4 | 366.63 | | |
| | Sample H19 | 28/5/2020 | 29.8 | 2922.4 | 332.09 | ***μ*** | ***σ*** |
| | Sample H20 | 28/5/2020 | 31.1 | 3049.9 | 346.58 | 342.1 | 19.0 |

## Water column pressure test H500

**Unsterilised material (from package)**

| | | | cm H20 | | |
|---|---|---|---|---|---|
| White Layer H500 | Sample PT-H500-1 | 28/05/2020 | 82 | | |
| | Sample PT-H500-2 | 28/05/2020 | 104 | | |
| | Sample PT-H500-3 | 28/05/2020 | 108 | ***μ*** | ***σ*** |
| | Sample PT-H500-4 | 28/05/2020 | 93 | 96.8 | 11.7 |
| Blue Layer H500 | Sample PT-H500-5 | 28/05/2020 | 76 | | |
| | Sample PT-H500-6 | 28/05/2020 | 81 | | |
| | Sample PT-H500-7 | 28/05/2020 | 83 | ***μ*** | ***σ*** |
| | Sample PT-H500-8 | 28/05/2020 | 83 | 80.8 | 3.3 |
| Single Layer H500 | Sample PT-H500-9 | 28/05/2020 | 129 | | |
| | Sample PT-H500-10 | 28/05/2020 | 124 | | |
| | Sample PT-H500-11 | 28/05/2020 | 128 | ***μ*** | ***σ*** |
| | Sample PT-H500-12 | 28/05/2020 | 121 | 125.5 | 3.7 |
| Double Layer H500 | Sample PT-H500-13 | 28/05/2020 | 128 | | |
| | Sample PT-H500-14 | 28/05/2020 | 142 | | |
| | Sample PT-H500-15 | 28/05/2020 | 129 | ***μ*** | ***σ*** |
| | Sample PT-H500-16 | 28/05/2020 | 146 | 136.3 | 9.1 |
| Triple Layer H500 | Sample PT-H500-17 | 28/05/2020 | 141 | | |
| | Sample PT-H500-18 | 28/05/2020 | 146 | | |
| | Sample PT-H500-19 | 28/05/2020 | 155 | ***μ*** | ***σ*** |
| | Sample PT-H500-20 | 28/05/2020 | 160 | 150.5 | 8.6 |
